# Prospectively measured triiodothyronine levels are positively associated with breast cancer risk in postmenopausal women

**DOI:** 10.1186/bcr2587

**Published:** 2010-06-11

**Authors:** Ada Tosovic, Anne-Greth Bondeson, Lennart Bondeson, Ulla-Britt Ericsson, Johan Malm, Jonas Manjer

**Affiliations:** 1 Department of Surgery, Skåne University Hospital Malmö, SE-205 02 Malmö, Sweden; 2 University and Regional Laboratories Region Skåne, Department of Pathology, Skåne University Hospital Malmö, SE-205 02 Malmö, Sweden; 3 Department of Endocrinology, Skåne University Hospital Malmö, SE-205 02 Malmö, Sweden; 4 Department of Laboratory medicine, Skåne University Hospital Malmö, SE-205 02 Malmö, Sweden; 5 The Malmö Diet and Cancer Study, Skåne University Hospital Malmö, SE-205 02 Malmö, Sweden

## Abstract

**Introduction:**

The potential association between hypo- and hyperthyroid disorders and breast cancer has been investigated in a large number of studies during the last decades without conclusive results. This prospective cohort study investigated prediagnostic levels of thyrotropin (TSH) and triiodothyronine (T3) in relation to breast cancer incidence in pre- and postmenopausal women.

**Methods:**

In the Malmö Preventive Project, 2,696 women had T3 and/or TSH levels measured at baseline. During a mean follow-up of 19.3 years, 173 incident breast cancer cases were retrieved using record linkage with The Swedish Cancer Registry. Quartile cut-points for T3 and TSH were based on the distribution among all women in the study cohort. A Cox's proportional hazards analysis was used to estimate relative risks (RR), with a confidence interval (CI) of 95%. Trends over quartiles of T3 and TSH were calculated considering a *P*-value < 0.05 as statistically significant. All analyses were repeated for pre- and peri/postmenopausal women separately.

**Results:**

Overall there was a statistically significant association between T3 and breast cancer risk, the adjusted RR in the fourth quartile, as compared to the first, was 1.87 (1.12 to 3.14). In postmenopausal women the RRs for the second, third and fourth quartiles, as compared to the first, were 3.26 (0.96 to 11.1), 5.53 (1.65 to 18.6) and 6.87 (2.09 to 22.6), (*P*-trend: < 0.001). There were no such associations in pre-menopausal women, and no statistically significant interaction between T3 and menopausal status. Also, no statistically significant association was seen between serum TSH and breast cancer.

**Conclusions:**

This is the first prospective study on T3 levels in relation to breast cancer risk. T3 levels in postmenopausal women were positively associated with the risk of breast cancer in a dose-response manner.

## Introduction

Thyroid disorders and breast cancer both have a postmenopausal peak incidence, and a potential association between hypo- and hyperthyroid disorders and breast cancer has been investigated in a large number of studies during the last decades [1-19]. However, the results have not been conclusive.

Experimental studies have shown that thyroid hormones can have estrogen-like effects in breast cancer, and that thyroid hormone receptors influence both normal breast cell differentiation and breast cancer cell proliferation [2,3]. Several clinical and epidemiological cross-sectional studies have been performed comparing levels of triiodothyronine (T3), thyroxin (T4) and thyrotropin (TSH) in breast cancer patients versus healthy controls. The results have been contradictory, some in favor of higher levels in cases [4-7] other in controls [8] and yet some report no differences [9-15].

It is, however, difficult to conclude from cross-sectional studies whether differences in thyroid hormone levels are associated with different risks of breast cancer, or if breast cancer itself alters thyroid hormone levels. To date, there is only one prospective cohort study on this issue, involving 61 breast cancer cases, where pre-diagnostic levels of TSH and T4 were related to subsequent risk of breast cancer [16]. The present study is a population based, prospective cohort study including 2,696 pre and peri/postmenopausal women in whom TSH and T3 levels were measured at baseline. During a follow-up of a total of 51,989 person-years, 173 women were diagnosed with incident breast cancer.

The aim of the present study was to investigate prediagnostic serum levels of TSH and T3 in relation to breast cancer incidence in pre- and peri/postmenopausal women, respectively.

## Materials and methods

### The Malmö Preventive Project

Originally, 10,902 women participated in the Malmö Preventive Project. The project was established in 1974 when residents in Malmö, a city in southern Sweden, were invited to participate in a health survey. Entire birth cohorts, men and women, were examined until 1992 when the department closed. Approximately 70% of invited subjects participated.

All participants answered a questionnaire concerning socio-demographic information, lifestyle habits, and medical history. Questions on reproductive factors, use of oral contraceptives (OC), and hormonal replacement therapy (HRT), were only included in women screened from April 1983 and onwards (8,051 subjects). There was no information on type of HRT. Body mass index (BMI) (kg/m2) was assessed by a trained nurse on baseline examination. A subject was considered to have a previous history of goiter if the question 'have you been treated for goiter' was answered with 'yes'. There was no available information on type of treatment.

The participants were initially part of a preventive health care project. All former participants were informed about the present study by newspaper as required by the local ethical committee. All participants were offered to be excluded from the present study.

### Triiodothyronine (T3) and thyrtropin (TSH) analysis

Blood samples were taken after an overnight fast with the patient in the supine position. The serum samples were analyzed for T3 and TSH in women born in 1928 and 1941 and examined in 1983 and 1984. In women born in 1935 (examined from 1990 to 1992), TSH levels were measured in all, but T3 was only measured in a sub-set of all women. In women born in 1935, T3 was analyzed in those with pathological TSH values, a history of thyroid disease, or those with an enlarged thyroid gland at examination. In addition to this, the attending physician could also decide to analyze T3. The basis for the decision to analyze T3 in an individual subject was not recorded systematically.

T3 was measured by a double anti-body radioimmunoassay (reference interval 0.9 to 3.2 mmol/l) [20]. Only six women had a value above the upper reference limit. TSH was also measured by a double anti-body radioimmunoassay [20] in 1983 and 1984 (period I, reference interval <8 mIU/l), whereas a immunoradiometric analysis (IRMA) was used in 1990 to 1992 (period II, reference interval 0.4 to 4.0 mIU/l). Data on coefficients of variation from the laboratory analysis were unfortunately not available.

### Study cohort

Among 10,902 women, reproductive data including menopausal status had been assessed in 8,051 subjets. Serum TSH had been measured in 2,944 of these (women born in 1928, 1941 and 1935, respectively). Those with prevalent breast cancer (n = 46), goiter (n = 196) or both of these conditions (n = 6) were identified and excluded from the study.

Finally, 2,696 women constituted our study population. Information on T3 was not available for all women born in 1935, and T3 had been assessed in 2,185 out of all 2,696 women. The present study was approved by the ethical committee at Lund University, Sweden: Dnr 652/2005 and Dnr 501/2006.

### Follow-up

Breast cancer cases, invasive and *in situ*, were retrieved up until 31 December 2006 by record linkage with The Swedish Cancer Registry (until 31 December 2005) and, due to a one-year delay in registration, also from its regional branch, The Southern Swedish Regional Cancer Registry (cases diagnosed in 2006). Among 2,696 women, there were 173 incident breast cancer cases. Information on vital status was retrieved from the Swedish Cause-of-Death Registry.

Mean follow-up was 19.3 (5.08) years and total follow-up was 51,989 person-years.

### Statistical methods

Quartile cut-points for T3 and TSH were based on the distribution among all women in the study cohort. Due to the change of method of analysis of TSH, separate cut-points were used for samples analyzed before and after this change.

Each woman was followed until the end of follow-up, 31 December 2006, or until she got breast cancer or died. The incidence of breast cancer per 100,000 person-years was calculated in different quartiles of T3 and TSH. A Cox's proportional hazards analysis was used to estimate corresponding relative risks (RR) with a confidence interval (CI) of 95%. Possible confounding factors, that is, established and potential risk factors for breast cancer, were introduced as covariates in a subsequent analysis. Age was entered as a continuous variable and all other factors were entered as categorical variables classified as in Tables 1 and 2 (menopausal status, use of HRT, number of children, use of oral contraception, age at menarche, smoking status, alcohol consumption, BMI, height, educational level and marital status). Trends over quartiles of T3 and TSH were calculated by introducing the quartile number as a continuous variable in the analysis. A *P*-value < 0.05 was considered as statistically significant.

**Table 1:** Distribution of potential risk factors for breast cancer according to serum T3 level

Factor	T3 quartile				
	
	1	2	3	4	All
	N = 494	N = 704	N = 456	N = 531	N = 2,185
	
	Column percent ***(T3 levels in italics)***				
T3 (mIU/l)	*<1.50*	*1.60 to 1.80*	*1.90 to 2.00*	*2.1 to -4.30*	
					
Age					
<50	62.1	46.6	26.1	21.5	39.7
>50	37.9	53.4	73.9	78.5	60.3
					
Premenstrual	60.3	44.2	28.7	23.2	39.5
Peri/postmenopausal	39.7	55.8	71.3	76.8	60.5
					
HRT in peri/postmenopausal	13.3	14.2	15.4	17.6	15.4
missing	-	-	0.3	-	0.1
					
Nulliparity	18.2	17.0	13.2	19.6	17.1
missing	-	-	0.4	0.8	0.3
					
OC-use	4.7	6.0	9.0	11.5	7.6
missing	-	-	0.7	0.9	0.4
					
Menarche <12 years	15.2	13.4	9.6	11.9	12.6
missing	-	0.4	1.3	1.3	0.7
					
Smoking					
never	43.5	47.7	47.6	47.1	46.6
current	35.8	33.2	34.0	33.1	34.0
ex	20.6	19.0	18.0	18.8	19.1
missing	-	-	0.4	0.9	0.3
					
Alcohol consumption					
nothing	14.2	14.1	13.2	19.0	15.1
less than every week	55.3	60.7	62.5	60.1	59.7
every week	30.6	25.1	23.9	19.4	24.7
missing	-	0.1	0.4	1.5	0.5
					
BMI					
<20	14.8	10.1	7.9	7.5	10.1
≥20 <25	62.3	56.1	53.9	46.5	54.7
≥25 <30	17.0	26.1	27.9	30.5	25.5
≥30	5.9	7.7	10.3	15.4	9.7
					
Height					
≤ 160	24.1	28.8	28.9	36.5	29.7
>160 ≤ 165	32.6	33.2	30.9	30.7	32.0
>165 ≤ 170	26.7	24.9	25.0	22.0	24.6
>170	16.6	13.1	15.1	10.7	13.7
					
Education					
<12 years	53.0	53.6	62.5	64.2	57.9
12 years	20.9	27.4	22.1	21.8	23.5
>12 years	21.7	14.9	11.2	10.7	14.5
Missing	4.5	4.1	4.2	3.2	4.0
					
Married	54.9	54.4	51.3	50.3	52.9
missing	0.8	1.8	7.2	14.5	5.8

Abbreviations: T3, triiodothyronine; HRT, hormone replacementtherapy; OC, oral contraceptives; BMI, body mass index.

**Table 2:** Distribution of potential risk factors for breast cancer according to serum TSH level

Factor	TSH quartile				
	
	1	2	3	4	All
	N = 647	N = 528	N = 793	N = 710	N = 2,678
	
	Column percent ***(TSH levels in italics)***				
TSH (mIU/l)					
period I	*<1.50*	*1.60 to 2.00*	*2.10 to 3.00*	≥*3.10*	
period II	*<0.90*	*1.00 to 1.30*	*1.40 to 2.00*	≥*2.10*	
					
Age					
<50	21.5	42.6	40.9	24.9	32.3
>50	78.5	57.4	59.1	75.1	67.7
					
Premenopausal	24.0	41.9	41.5	27.3	33.6
Peri/postmenopausal	76.0	58.1	58.5	72.7	66.4
					
HRT in peri/postmenopausal	17.3	20.8	17.2	13.0	16.6
missing	0.2	-	-	-	0.1
					
Nulliparity	13.9	13.1	18.0	18.2	16.1
missing	1.4	1.9	0.5	0.3	0.9
					
OC-use	3.7	8.7	8.7	4.4	6.3
missing	1.7	2.1	0.8	0.3	1.1
					
Menarche <12 years	13.4	12.5	13.0	12.0	12.7
missing	2.3	3.6	1.0	0.6	1.7
					
Smoking					
never	42.2	41.9	47.9	51.0	46.2
current	36.0	36.9	31.9	29.7	33.3
ex	20.2	19.3	19.7	18.9	19.5
missing	1.5	1.9	0.5	0.4	1.0
					
Alcohol consumption					
nothing	17.0	12.3	14.2	16.5	15.1
less than every week	59.4	53.2	57.8	60.0	57.8
every week	21.5	30.7	26.5	23.0	25.2
missing	2.2	3.8	1.5	0.6	1.9
					
BMI					
<20	10.0	11.6	9.1	7.3	9.3
≥20 <25	53.3	50.8	57.9	52.0	53.8
≥25 <30	26.9	27.3	23.8	28.9	26.6
≥30	9.7	10.4	9.2	11.8	10.3
					
Height					
≤ 160	30.3	26.9	29.5	33.9	30.4
>160 ≤ 165	32.8	29.5	31.8	33.2	32.0
>165 ≤ 170	26.0	27.5	25.3	21.7	24.9
>170	11.0	16.1	13.4	11.1	12.7
					
Education					
<12 years	58.6	54.4	55.1	61.0	57.4
12 years	23.3	24.6	26.4	22.0	24.1
>12 years	14.2	18.8	14.2	13.2	14.9
missing	3.9	2.3	4.3	3.8	3.7
					
Married	45.0	49.6	51.2	54.9	50.4
missing	19.6	25.0	7.6	4.2	13.0

Abbreviations: TSH, thyrotropin; HRT, hormone replacementtherapy; OC, oral contraceptives; BMI, body mass index.

Breast cancer may be associated with different risk factors in peri/postmenopausal versus premenopausal women. Furthermore, both thyroid conditions and breast cancer have been associated with BMI [21,22]. Following this, all analyses were repeated for pre- and peri/postmenopausal women separately, and stratified for BMI (BMI <25 vs. BMI ≥25). The potential interaction between T3/TSH and menopausal status was tested by adding an interaction term in the Cox analysis. A *P*-value <0.05 was considered indicative of a statistically significant interaction.

It has been reported that the relation between TSH and T3/T4 may be disturbed in breast cancer patients, hence the presence of undiagnosed breast cancer may affect thyroid hormone levels [16,23,24]. Consequently, cases diagnosed within three years after baseline examination and subjects with less than three years follow-up were excluded in an additional analysis. As it is possible that certain combinations of T3/T4 and TSH can be related to an increased risk of breast cancer, different combinations of low (below median) and high (above median) TSH and T3 were examined in relation to risk of breast cancer. Moreover, the risk of breast cancer in relation to T3 quartiles was analyzed in different strata of TSH (below and above the median). Interaction between T3 and TSH was investigated by entering an interaction term in the Cox analysis. T3 was entered as quartiles, and TSH as a dichotomies variable (below/above median).

Since T3 was not measured in the whole group born in 1935, the analysis of T3 was repeated including only women born in 1928 and 1941.

Due to the potential effect of estrogens on thyroxin binding globulin (TBG) concentration, and hence the levels of T3, an additional analysis was executed excluding women treated with HRT or OC.

## Results

Advanced age, peri/postmenopausal status, use of OC, HRT and high BMI, were more common in high T3 quartiles (Table 1). With regard to TSH, the highest percentage of peri/postmenopausal women, were in the lowest and highest TSH quartiles, respectively (Table 2).

Overall, the risk of breast cancer was statistically significantly higher in the fourth T3 quartile as compared to the first quartile (Table 3). This association was even stronger for postmenopausal women, also showing a marked dose-response pattern (*P*-value for trend < 0.001). No such associations were seen in premenopausal women. There were no statistically significant interactions between menopausal status and T3 (*P*: 0.48) or TSH (*P*: 0.83).

**Table 3:** Breast cancer incidence in pre-, peri/postmenopausal and all women in relation to serum T3 levels

Group	T3 (quartile)	Individuals (n)	Breast cancer cases (n)	Person-years	Incidence/100,000	All cases		Cases >3 years following Baseline examination	
							
						RR (CI: 95%)	RR** (CI: 95%)	RR (CI: 95%)	RR** (CI: 95%)
All	1	494	28	10,418	269	1.00	1.00	1.00	1.00
	2	704	40	14,613	274	1.02 (0.63 to 1.66)	1.12 (0.69 to 1.84)	1.10 (0.66 to 1.83)	0.99 (0.60 to 1.66)
	3	456	31	9,096	341	1.28 (0.77 to 2.13)	1.42 (0.83 to 2.43)	1.37 (0.78 to 2.42)	1.21 (0.70 to 2.10)
	4	531	47	10,091	466	1.75 (1.10 to 2.80)*	1.87(1.12 to 3.14)*	1.98 (1.15 to 3.38)*	1.78 (1.10 to 2.90)*
	*P*-trend					0.007	0.009	0.007	0.008
Premenopausal	1	298	25	6,376	392	1.00	1.00	1.00	1.00
	2	311	21	6,621	317	0.81 (0.45 to 1.45)	1.00 (0.55 to 1.81)	1.00 (0.53 to 1.87)	0.80 (0.43 to 1.46)
	3	131	7	2,816	249	0.63 (0.27 to 1.47)	0.71 (0.30 to 1.70)	0.79 (0.33 to 1.91)	0.69 (0.33 to 1.61)
	4	123	9	2,610	345	0.88 (0.41 to 1.88)	0.92 (0.40 to 2.15)	1.07 (0.45 to 2.50)	0.96 (0.44 to 2.10)
	*P*-trend					0.49	0.66	0.94	0.99
Peri/post-menopausal	1	196	3	4,042	74	1.00	1.00	1.00	1.00
	2	393	19	7,992	237	3.24 (0.96 to 10.9)	3.26 (0.96 to 11.1)	2.75 (0.80 to 9.48)	2.90 (0.85 to 9.91)
	3	325	24	6,281	382	5.21 (1.57 to 17.4)*	5.53 (1.65 to 18.6)*	4.63 (1.35 to 15.8)*	4.39 (1.30 to 14.8)*
	4	408	38	7,481	508	6.94 (2.14 to 22.5)*	6.87 (2.09 to 22.6)*	6.14 (1.86 to 20.3)*	6.49 (1.99 to 21.1)*
	*P*-trend					<0.001	<0.001	<0.001	<0.001

Abbreviations: T3, triiodothyronine; RR, relative risk; RR**, relative risk adjusted for age (continuous), menopausal status (in analysis of all women), use of HRT, nulliparity, use of oral contraception, age at menarche, smoking status, alcohol consumption, BMI, height, educational level and marital status, * *P *< 0.05.

There was no overall statistically significant association between serum TSH levels and breast cancer (Table 4).

**Table 4:** Breast cancer incidence in pre-, peri/postmenopausal and all women in relation to serum TSH levels

Group	TSH (quartile)	Individuals (n)	Breast cancer cases (n)	Person-years	Incidence/100,000	All cases		Cases >3 years following Baseline examination	
							
						RR (CI: 95%)	RR** (CI: 95%)	RR (CI: 95%)	RR** (CI: 95%)
All	1	647	40	11,764	340	1.00	1.00	1.00	1.00
	2	528	42	9,517	441	1.05 (0.70 to 1.58)	1.07 (0.71 to 1.62)	1.28 (0.81 to 2.01)	1.26 (0.80 to 1.97)
	3	793	55	15,829	347	0.93 (0.62 to 1.41)	0.94 (0.62 to 1.42)	1.20 (0.77 to 1.87)	1.19 (0.76 to 1.85)
	4	710	36	14,521	248	0.87 (0.55 to 1.37)	0.80 (0.50 to 1.27)	0.95 (0.57 to 1.57)	1.03 (0.62 to 1.69)
	*P*-trend					0.46	0.29	0.82	0.94
Premenopausal	1	155	10	3,170	315	1.00	1.00	1.00	1.00
	2	221	18	4,669	386	1.48 (0.76 to 2.90)	1.38 (0.69 to 2.77)	1.91(0.90 to 4.08)	2.02 (0.97 to 4.23)
	3	329	28	6,869	408	0.95 (0.48 to 1.87)	0.94 (0.47 to 1.87)	1.31(0.62 to 2.79)	1.30 (0.62 to 2.72)
	4	194	7	4,213	166	0.81 (0.36 to 1.86)	0.62 (0.26 to 1.46)	0.88 (0.35 to 2.21)	1.11 (0.46 to 2.67)
	*P*-trend					0.42	0.20	0.64	0.91
Peri/post-menopausal	1	492	30	8,593	349	1.00	1.00	1.00	1.00
	2	307	24	4,848	495	0.86 (0.51 to 1.45)	0.87 (0.51 to 1.47)	0.93 (0.52 to 1.67)	0.94 (0.53 to 1.67)
	3	464	27	8,960	301	0.93 (0.56 to 1.56)	0.94 (0.56 to 1.58)	1.13 (0.65 to 1.98)	1.14 (0.66 to 1.99)
	4	516	29	10,308	281	0.88 (0.51 to 1.53)	0.82 (0.47 to 1.43)	0.89 (0.48 to 1.65)	0.97 (0.53 to 1.78)
	*P*-trend					0.73	0.57	0.94	0.87

Abbreviations: TSH, thyrotropin; RR, relative risk; RR**, relative risk adjusted for age (continuous), menopausal status (in analysis of all women), use of HRT, nulliparity, use of oral contraception, age at menarche, smoking status, alcohol consumption, BMI, height, educational level and marital status. * *P *< 0.05.

In the analyses stratified for BMI, there was a weak, non-significant association between high T3 levels and breast cancer in the group with BMI over 25, the RR for the fourth vs. the first quartile was 2.41 (0.99 to 5.84), as compared to the corresponding RR in women with BMI below 25, 1.31 (0.72 to 2.41) (other data not shown).

Adjusted RRs were very similar to crude RRs with regard to both T3 and TSH. When women who developed breast cancer within three years after screening were excluded from the analyses, all results were similar for T3, but for TSH in premenopausal women, the risk in the second quartile was even stronger (Tables 3 and 4).

The analyses of combinations of low and high T3/TSH levels showed a statistically significant association for crude RR in the highT3/highTSH groups, as compared to the low/low category (1.62: 1.01 to 2.60). The association was, however, not statistically significant in the adjusted analysis (1.63: 0.99 to 2.69). The data were also stratified for TSH, below and above median with regard to T3 quartiles. There was a statistically significant risk for breast cancer in the highest T3 quartile (2.69: 1.19 to 6.08) in women with a TSH above the median. The corresponding RR in women with low TSH levels was 1.62 (0.68 to 3.86). There was no statistically significant interaction between T3 and TSH in the whole cohort, in premenopausal or peri/postmenopausal women.

When the analyses were repeated, excluding the women born in 1935, all results were similar, crude RR = 5.56 (1.66 to 18.57) for postmenopausal women in the highest T3 quartile (other data not shown).

Also, the analyses were repeated excluding women treated with HRT and OC, and the results were similar, crude RR = 6.50 (1.99 to 21.2) for postmenopausal women in the highest T3 quartile (other data not shown).

## Discussion

This is the first prospective study on T3 levels in relation to breast cancer risk. It shows that T3 levels in postmenopausal women are positively associated with the risk of breast cancer in a dose-response manner.

It may be asked whether breast cancer cases in this cohort are representative of the whole breast cancer population. This cohort mainly comprised middle-aged women and 70% of the women invited to the health examination attended. As we have no information about exposure to the studied risk factors in women outside this cohort, observed incidence rates may not be applicable to all age groups or to the general population. However, as there was a wide distribution of T3 and TSH levels, it was possible to make internal comparisons between subjects with low and high values, respectively. We consider that our estimations of relative risks were not considerably affected by selection bias.

Incomplete follow-up may affect the results. However, the Swedish Cancer Registry and the Swedish Cause-of-Death Registry have been validated and found to have a completeness of about 99% [25].

Subjects with elevated T3 levels and a possible diagnosis of hyperthyroidism may already be within the health care system and due to this the diagnosis of breast cancer might be established earlier than for euthyroid subjects. However, general mammography screening was introduced in Sweden already in 1986 and stands for the majority of detection of breast cancer cases [26]. It is unlikely that the thyroid status does affect participation in mammography screening, hence a possible detection bias in hyperthyroid breast cancer patients was probably not important. It should also be noted that most women with T3 levels in the fourth quartile in the present study still had levels within the normal range, and these women did probably not have any symptoms of thyroid disease.

The method of analysis for T3 remained the same throughout the study. Not all women in the study population had information on T3, but our analyses showed no difference in risk of breast cancer in relation to T3 when the group screened in 1990 was excluded. Hence, there was probably no major selection bias due to the inclusion of only some subjects with information on T3 in the later period.

The method of analyses for TSH changed between 1983 and 1990 as did the reference values. This was taken into account in our statistical analysis.

There is a well known circadian and seasonal variation concerning T3 and TSH levels [27]. Tracking of individuals, that is, ranking of individuals over time is, however, quite stable [28]. A true variation over time would most likely have led to a un-differential misclassification of T3 and TSH, and, hence, attenuated observed risks.

The use of total T3 as a marker of T3 status involves an important methodological aspect. Most T3 in the circulation is bound to three transport proteins, thyroxin binding globulin (TBG), transthyretin and albumin. An increased TBG concentration leads to higher levels of total T3, and this is important to consider in studies of breast cancer risk, as exogenous estrogens, that are risk factors for breast cancer, that is, HRT and OC, led to increased TBG binding capacity [29]. In order to exclude the association between total T3 levels and breast cancer risk as only an effect of differences in the use of HRT and OC, we adjusted our analyses for HRT and OC. Moreover, the analyses in postmenopausal women were repeated excluding women that had used HRT or OC. Following that exclusion, the RR for T3, as compared to the first quartile, was in the second quartile 3.11 (0.92 to 10.6), in the third 4.55 (1.35 to 15.3) and in the fourth quartile 6.50 (1.99 to 21.2). Hence, both adjustment for HRT/OC and exclusion of subjects using these medications resulted in similar risk estimates as were seen in the main analysis. This finding, together with the notion that there is no known association between endogenous estrogen levels and TBG [29], makes it unlikely that the results in the present study were caused by differences with regard to TBG levels.

In the questionnaire used to collect data from the participants, there was no information on co-morbidities or medication which may affect the thyroid function, for example, propanolol, salicylates and phenytoin. This is a limitation in the study, but as there is no known association between such factors and breast cancer risk, the effect from uncontrolled confounding was probably small. There was no information on thyroxin use specifically in the questionnaire, which is another limitation. This was handled by excluding women who affirmed any treatment for *goiter*, which probably limited the interference with the results in the present study. However, the current material did not allow any assessment of the risk of breast cancer following thyroxin therapy.

The current study is the largest prospective study to date on T3/TSH levels and breast cancer risk, but the number of cases was limited. Confidence intervals were wide and the statistical power relatively low. This led to an imprecision in the estimates related to T3, and there is a risk that the lack of association between TSH and breast cancer was due to a type II error.

A large number of epidemiological studies have been performed during the last 50 years on benign thyroid conditions in relation to breast cancer [9-14,30-32], and at least seven studies included more than 1,000 breast cancer cases [9-14,30]. However, most previous studies show no association between thyroid condition and breast cancer. A methodological problem is that the great majority of these studies were cross-sectional; hence it may be difficult to establish a potential causal relationship between thyroid disorders and breast cancer. A study by Cristofanilli *et al*., including 1,136 breast cancer cases, reported that women with previous hypothyroid conditions had an increased risk of breast cancer [30]. Another prospective study including a cohort of 2,775 women found that previous hypothyroidism and the use of thyroid medications was associated with an odds ratio of 3.8 for breast cancer [16]. An indirect evidence of an association between hypothyroidism and breast cancer is offered by studies following women treated for thyroid cancer (that is, by surgery or radio isotopes causing a hypothyroid state). For example, Brown *et al*. used the SEER registry and found an increased risk of breast cancer in women with a previous diagnosis of thyroid cancer [33]. These three studies were prospective, but a methodological problem is that the majority of these patients had been treated with thyroxin. That is, the results may have been confounded by exposure to exogenous thyroid hormones. Moreover, as the above studies used information on thyroid disorders usually obtained by interviews or record-linkage with diagnosis registries thyroid hormone levels were not directly measured. Thus, these studies do not rule out the possibility that sub-clinical hyperthyroid conditions, or even elevated T3 levels within the normal range, may play a role in breast cancer pathogenesis.

Some previous cross-sectional studies have found that high T3 levels are positively associated with breast cancer [1,5,7], among them the largest study to date including 226 cases (mean age 55 years, range 28 to 79) and 166 controls (mean age 46 years, range 17 to 65) [5]. Turken *et al*. examined 150 cases and 100 controls, and the results indicated that high T4 levels were associated with breast cancer [4]. On the other hand, several reports have suggested an association between low T3 [8,24,34] and/or low T4 levels [8] and breast cancer risk. Indeed, the only prospective study found a negative association between T4 levels and breast cancer risk [16]. Finally some studies did not show any associations at all with regard to T3/T4 and breast cancer [17,35,36], the two largest with 356 and 136 cases, respectively [35,7]. High TSH levels have been associated with breast cancer risk in some studies [24,37], but the only prospective analysis to date found a negative association [16]. Yet, most have found no association between TSH levels and breast cancer [3,5,7,8,35,36,38-40]. One study found that TSH is low in cases among postmenopausal women but high in cases among premenopausal [1], indicating that menopause status may modify the potential association between thyroid hormones and risk of breast cancer. Autoimmune thyroid diseases (defined as the presence of thyroid peroxidase antibodies; TPOab) have been positively associated with breast cancer risk in several cross-sectional studies [4,17,40]. It is unclear whether the presence of TPOab in serum from patients with breast cancer is related to an increased risk following TPOab related conditions or if it is a general autoimmune response to the malignancy [41]. Indeed, the only prospective study showed that the pre-diagnostic presence of TPOab was not related to the subsequent risk of breast cancer [16]. In our prospective study, breast cancer cases diagnosed within three years after screening were excluded, hence the effect of (subclinical) cancer disease on thyroid hormones or TSH, that is, reverse causality, is unlikely.

The presence of thyroid hormone receptors in human breast and breast cancer cell lines has been established [42-45], and the proliferative effect of T3 has been confirmed by various experimental studies on breast cancer. Our findings are in line with these data [1-3,46].

It has been shown that T3 binds and stimulates the estrogen receptor, acting in synergy with estrogen on breast cancer cell lines, potentiating the estrogenic effect and enhancing cell proliferation [47]. The role of estrogen in carcinogenesis of the breast is well known and the possibility that this effect may be even stronger in conjunction with high levels of T3 could in part explain the results in the present study.

Obesity is associated with increased breast cancer risk in postmenopausal women [48]. Estrogen levels are higher in obese compared to normal weight women. The potentiating effect of T3 might further increase the risk in this subgroup. Although our results did not reach statistical significance, they are in line with this hypothesis.

The significant positive association between T3 and breast cancer is specifically strong in postmenopausal women. Therefore, an imbalance between estrogen and T3 with an increased T3/E2 ratio may be more important than a pure synergistic effect between these two hormones for the risk of developing breast cancer. It has been suggested that this imbalance might enhance breast cancer development [1]. This theory is in accordance with our findings that higher T3 levels in postmenopausal women are positively associated with the risk of breast cancer in a dose-response manner. The present study excluded women with a previous treatment for goiter and there was no information on thyroxin use. An important issue for future studies will be to evaluate the widespread, long-term use of thyroxin treatment in TSH suppressive doses for benign thyroid disease [49,50], and the management of women with subclinical hyperthyroidism [51-53]. Such studies will contribute with important evidence whether or not to treat mild and/or sub-clinical thyroid disorders.

## Conclusions

In conclusion, the present prospective cohort study, the first of its kind on prospectively measured T3 levels and breast cancer risk, indicates that high T3 levels in postmenopausal women are positively associated with the risk of breast cancer in a dose-response manner.

## Abbreviations

BMI: body mass index; CI: confidence interval; E2: estrogen; HRT: hormone replacement therapy; IRMA: imunnoradiometric analysis; OC: oral contraceptives; RR: relative risk; T3: triiodohyronine; T4: thyroxin; TBG: thyroxin binding globulin; TPOab: thyroid peroxidase antibodies; TSH: thyrotropin.

## Competing interests

The authors declare that they have no competing interests.

## Authors' contributions

AT and JM made substantial contributions to the conception and design of the study. AT, JM and UBE contributed to the analysis of and interpretation of data. UBE and JM contributed to the acquisition of data. All authors were involved in drafting the manuscript and revising it critically for important intellectual content. All authors read and approved the final manuscript.
